# The Role of Sodium Bicarbonate in the Management of Some Toxic Ingestions

**DOI:** 10.1155/2017/7831358

**Published:** 2017-08-08

**Authors:** Aibek E. Mirrakhimov, Taha Ayach, Aram Barbaryan, Goutham Talari, Romil Chadha, Adam Gray

**Affiliations:** ^1^Department of Medicine, University of Kentucky College of Medicine, Lexington, KY, USA; ^2^University of Kansas Medical Center, Kansas City, KS, USA

## Abstract

Adverse reactions to commonly prescribed medications and to substances of abuse may result in severe toxicity associated with increased morbidity and mortality. According to the Center for Disease Control, in 2013, at least 2113 human fatalities attributed to poisonings occurred in the United States of America. In this article, we review the data regarding the impact of systemic sodium bicarbonate administration in the management of certain poisonings including sodium channel blocker toxicities, salicylate overdose, and ingestion of some toxic alcohols and in various pharmacological toxicities. Based on the available literature and empiric experience, the administration of sodium bicarbonate appears to be beneficial in the management of a patient with the above-mentioned toxidromes. However, most of the available evidence originates from case reports, case series, and expert consensus recommendations. The potential mechanisms of sodium bicarbonate include high sodium load and the development of metabolic alkalosis with resultant decreased tissue penetration of the toxic substance with subsequent increased urinary excretion. While receiving sodium bicarbonate, patients must be monitored for the development of associated side effects including electrolyte abnormalities, the progression of metabolic alkalosis, volume overload, worsening respiratory status, and/or worsening metabolic acidosis. Patients with oliguric/anuric renal failure and advanced decompensated heart failure should not receive sodium bicarbonate.

## 1. Introduction

In the USA, at least 34% of the population regularly takes at least one prescription medication [1]. In the vast majority of cases the use of such prescribed medications provides clinical benefit to patients. However, in some cases, these medications may result in harm including a severe illness and death due to intentional or unintentional overdose or as a result of idiosyncratic drug reaction.

The goal of this article is to review the data and evidence on the use of sodium bicarbonate in the management of some pharmacological overdoses. We will first briefly review the mechanisms of metabolic acidosis related biochemical derangements since some of the overdoses are associated with metabolic acidosis. Second, we will discuss the mechanism of action, potential side effects, and typical dosing of sodium bicarbonate. Third, we will review the literature on the role of sodium bicarbonate in the management of sodium channel blocker toxicities. Fourth, the data on the role of sodium bicarbonate in the management of salicylate poisoning will be provided. Finally, we will discuss the place of sodium bicarbonate in the management of toxic alcohol ingestions.

The management of the toxicities mentioned above is complicated, requiring additional measures than sodium bicarbonate alone. Additional information on other aspects of management can be obtained elsewhere and is not the goal of this article.

## 2. Mechanism of Action and Potential Complications of Sodium Bicarbonate Therapy

Bicarbonate is an essential chemical regulating the acid-base balance acting as a buffer [2]. Carbon dioxide (CO_2_) is a major byproduct of energy metabolism in living organisms and a conjugate acid. Carbonic anhydrase enzyme facilitates the chemical interaction between CO_2_ and water producing carbonic acid (H_2_CO_3_). Carbonic acid in turn dissociates into bicarbonate (HCO_3_^−^) and hydrogen ion (H^+^) with the latter being removed via kidneys. HCO_3_^−^ can also bind extra H^+^ producing carbonic acid. Indeed, all the reactions are reversible and can go in any direction.

**Figure 1:**
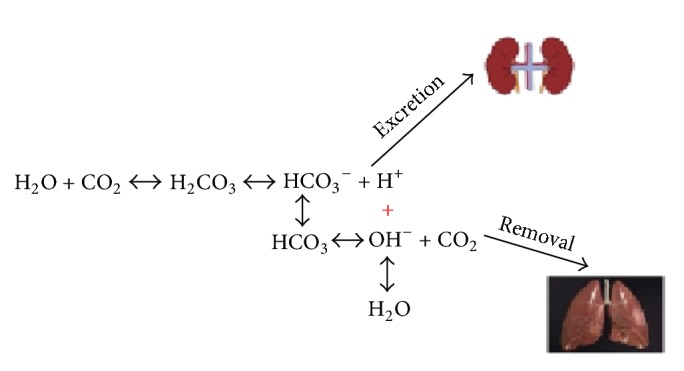
An overview of the endogenous bicarbonate metabolism.

Sodium bicarbonate (NaHCO_3_) is one of the few pharmacological agents aiming to mimic the endogenous effects of HCO_3_^−^. Sodium bicarbonate is widely used in many clinical situations including cardiac arrest [3, 4] and prevention of contrast-induced renal failure [5] and in patients with different types of metabolic acidosis (such as lactic acidosis and diabetic ketoacidosis) [6], despite limited and controversial evidence of its benefits. A simplified view on the bicarbonate chemistry is provided in Figure 1. An overview of chemical characteristics of commonly used crystalloids is presented in Table 1.

The potential benefits of exogenous intravenous sodium bicarbonate include the correction of metabolic acidosis with its associated detrimental effects. However, the role of sodium bicarbonate in the management of acute acidosis remains controversial and may even be associated with potential side effects and complications such as volume overload, metabolic alkalosis, hypercapnia, hypokalemia, hypernatremia and hyperosmolality, and ionized hypocalcemia [6, 7].

When administered, sodium bicarbonate dissociates into a molecule of sodium and bicarbonate. The molecule of bicarbonate in turn binds hydrogen converting into carbonic acid with its subsequent dissociation into carbon dioxide and water.

Carbon dioxide under normal conditions (intact lung perfusion and ventilation) is exhaled, maintaining delicate acid-base equilibrium. Under extreme conditions such as shock states and impaired ventilation, carbon dioxide may accumulate, leading to worsening acidosis [8, 9]. CO_2_ penetrates cellular membranes easily, and through this ability may exacerbate intracellular acidosis. Furthermore, by correcting the systemic acidosis, sodium bicarbonate administration may reduce respiratory drive leading to accumulation of CO_2_ in the central nervous system and associated adverse neurological sequelae [10]. Serum potassium may decrease as a result of potassium shift into the cells in the patient with metabolic acidosis treated with sodium bicarbonate. Other potential adverse effects of administered sodium bicarbonate may be related to its chemical features such as supraphysiologic sodium content and osmolality and alkaline pH (comparative chemical features of sodium bicarbonate and commonly used crystalloid solutions to plasma is presented in Table 1). Ionized calcium may be decreased, such as in metabolic alkalosis [11], which may cause tetany, decrease cardiac contractility, and potentially predispose to cardiac arrhythmias via prolongation of QT interval [12]. Serum sodium and osmolality tend to increase which may lead to cellular dehydration and systemic hypervolemia [13]. Lactic acid production may be increased in certain situations via alkalosis dependent activation of 6-phosphofructokinase enzyme [11, 14], and ketone bodies production may be enhanced [15]. Decreased cardiac oxygen supply and arterial vasoconstriction may also occur secondary to metabolic alkalosis [16, 17]. Lastly, serious skin injuries can occur in the setting of extravasation of hypertonic bicarbonate solutions, and whenever possible it should be administered through large bore intravenous lines or central venous lines.

Nevertheless, these adverse effects of sodium bicarbonate such as an increase in systemic pH and high sodium load may be useful in the management of certain pharmacological toxicities and overdoses. Below we will review the utility and likely mechanisms of sodium bicarbonate in the management of certain pharmacological toxicities: sodium channel blockers, salicylates, methanol and ethylene glycol poisonings, and, finally, miscellaneous pharmacological toxicities.

## 3. The Role of Sodium Bicarbonate in the Management of Sodium Channel Blocker Toxicities

Sodium channels are essential ion channels responsible for transcellular sodium influx, primarily in cardiac and neurological tissue [18]. Pharmacological agents targeting sodium channels are used in cardiac electrophysiology and neurology (particularly in the areas of pain management and epilepsy) [19, 20], as well as depression [21]. However, in cases of excessive administration (intentional or not), sodium channel blockade may lead to serious cardiac dysfunction. A list of some of the commonly used medications possessing sodium channel blocking activities is presented in Table 2.

Tricyclic antidepressants (TCAs) are among the oldest antidepressant medications that may also be used in the management of mood disorders, generalized anxiety disorder, panic disorder, and neuropathic pain [21]. Despite being an effective class, it is notorious for its narrow therapeutic index and major side effect profile in cases of overdose. TCAs were responsible for approximately 58% of all antidepressant medications related fatal toxicities in 2013 [22]. The pathogenesis of TCA toxicity in regard to sodium channel blockade is fundamental to the understanding of other sodium channel toxicities, as is the therapeutic role of intravenous (IV) sodium bicarbonate. It is important to remember that the pharmacopathogenesis of TCA toxicities is more complex than just sodium channel blockade and also includes inhibition of muscarinic, alpha-1 adrenergic, and antihistamine receptors [23]. Inhibition of cardiac sodium channels may manifest on the electrocardiogram (ECG) as the prolongation of QRS interval, new onset right axis deviation, deep S wave in lead AVL, tall R wave, and increased R wave to S wave ration in lead AVR and Brugada-like pattern [24–28]. Clinically the effects of sodium channel blockade may present as cardiac arrhythmias and hemodynamic instability [29]. Metabolic acidosis that occurs in cases of hemodynamic deterioration potentiates the sodium blocking activity of TCA medications by increasing the binding to sodium channels [29–31]. Furthermore, TCA toxicity can cause seizures resulting in persistence of metabolic acidosis and propagation of detrimental metabolic disturbances. TCA toxicity may be delayed and patients may initially appear clinically well until decompensation occurs.

The scientific data on the use of sodium bicarbonate in the management of TCA toxicity is predominantly originated from animal studies, case reports, and case series [32, 33]. Sodium bicarbonate is commonly administered as 8.4% solution 1-2 mEq/kg (see Table 1) in cases of TCA associated ECG abnormalities (such as QRS prolongation > 100 msec), hemodynamic compromise, and malignant ventricular arrhythmias [34–36]. The benefit of sodium bicarbonate in the setting of TCA overdose is probably due to both an increase in serum pH and the increase in extracellular sodium. Alkalization favors the neutral form of the drug and reducing the amount of active cyclic antidepressants. The high sodium load increases the electrochemical gradient across cardiac cell membranes, potentially attenuating the TCA-induced blockade of sodium channels [29, 30].

**Figure 2:**
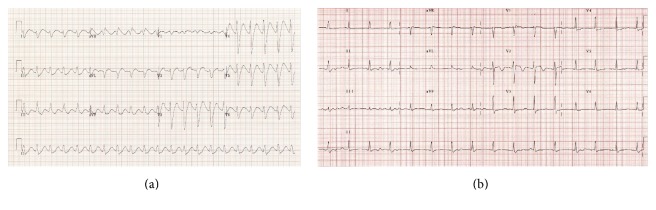
(a)* Sodium channel toxicity on 12-lead ECG (taken from https://lifeinthefastlane.com).* On this 12-lead ECG you can see regular wide QRS tachycardia with no clear P waves, right axis deviation, tall R wave in AVR, and poor R wave progression in precordial leads. (b)* Sodium channel toxicity on 12-lead ECG after administration of sodium bicarbonate (taken from https://lifeinthefastlane.com).* On this 12-lead ECG after administration of sodium bicarbonate you can see atrioventricular dissociation with normal QRS, normal axis, and resolution of tall R wave in AVR.

Based on clinical experience and available literature, the majority of patients tend to respond via improvement in hemodynamic and ECG parameters. 12-lead ECG of sodium channel blocker toxicity before and after administration of sodium bicarbonate is presented in Figures 2(a) and 2(b).

It may be necessary to continue sodium bicarbonate after bolus doses in the form of IV infusion by diluting 2-3 ampules in 1 liter of dextrose 5% solution that is nearly isotonic to plasma to decrease the risk of potential rebound deterioration, while, on IV sodium bicarbonate, patients should be monitored for evidence of fluid overload, respiratory status with advanced airway management when indicated, electrolyte abnormalities (hypernatremia, hypokalemia, hypocalcemia), and metabolic alkalosis (potential target pH of 7.5). It is important to keep in mind that because of its anticholinergic properties, the absorption of the TCA may be delayed (due to delayed gastrointestinal motility); these patients should be closely monitored, and, in cases of reemerging symptoms, sodium bicarbonate and other therapies must be timely administered. Patients treated with sodium bicarbonate should be monitored in the intensive care setting with continuous monitoring and reassessment. Frequency of laboratory testing and electrocardiographic monitoring should be individualized. Blood gas analysis (arterial or venous) and chemistry tests should be monitored at least every 4 hours or more frequently if clinically indicated in patients treated with sodium bicarbonate. It is important to keep calcium and potassium within normal range and replete them if low to decrease the risk of cardiac arrhythmias.

Other medications such as antiarrhythmics [36–38], non-TCA antidepressants [39, 40], antiepileptic medications [41–43], cyclobenzaprine [43, 44], propranolol [45], cocaine [46], and certain antihistamines [47] may produce similar ECG and clinical manifestations with favorable response to similarly administered IV sodium bicarbonate. Amantadine overdose may result in a similar cardiac sodium channel toxicity though IV sodium bicarbonate was not specifically used in the reported cases because of concomitant hypokalemia [48]. Animal research supports the beneficial effects of sodium bicarbonate in case of thioridazine toxicity [49].

In conclusion, the literature on the use of sodium bicarbonate in the management of sodium channel blocker toxicities is limited to animal research and case series; randomized trials are precluded due to ethical reasons. IV sodium bicarbonate should be considered in the cases of suspected sodium channel blocker toxicity associated with hemodynamic and ECG abnormalities, given the very high risk of adverse outcome without aggressive treatment [50]. Nevertheless, it is important to keep in mind that IV sodium bicarbonate represents only a single tool in the management of these toxicities and additional mechanisms of toxicity may account for protean clinical manifestations of these poisonings.

## 4. The Role of Sodium Bicarbonate in the Management of Salicylate Toxicity

Salicylates are a group of pharmacological agents that include aspirin, bismuth salicylate, and local skin preparations such as salicylic acid and methyl salicylate (topical preparations that rarely cause toxicity if used in an excessive amount or in patients with skin damage leading to increased absorption) [51, 52]. Aspirin, which is a major representative of the class, has myriad of indications including cardiovascular diseases, rheumatic diseases, and analgesia [53].

Analgesics, including aspirin, were the most common etiology of all drug poisonings in the USA, with salicylate poisoning leading to a fatal outcome in 34 patients out of 2113 deaths reported in 2013 [54]. The major mechanism of action of aspirin is mediated via inhibition of cyclooxygenase enzyme, resulting in decreased production of thromboxane A2 and various prostaglandins [53]. However, with higher dosages, other biochemical alterations may occur such as uncoupling of oxidative phosphorylation in the electron transport chain, resulting in heat release and stimulation of respiratory center in the medulla [23]. The decrease in the blood pH will favor formation of lipid soluble salicylic acid which easily penetrates blood-brain barrier and undergoes renal reabsorption [55].

Patients with salicylate toxicity typically present with tinnitus, gastrointestinal complications (nausea, vomiting, bleeding, and liver toxicity), hyperthermia (via uncoupling of oxidative phosphorylation), pulmonary edema, and mixed acid-base disorder (high anion gap metabolic acidosis and respiratory alkalosis via stimulation of respiratory center in the brain stem) [55].

Alkalinization with sodium bicarbonate is an essential component of management of the aspirin-poisoned patient. The first report in the English language medical literature of the positive effect of sodium bicarbonate in the management of salicylate-poisoned patient originates in 1948 [55].

Salicylic acid (HS) is a weak acid that exists in a charged (deprotonated, sal^−^) and uncharged (protonated, H^+^) form: H^+^ + sal^−^  *⇔* HS. Uncharged molecules (HS), unlike charged molecules (sal^−^), can move easily across cellular barriers, including the blood-brain barrier and the epithelium of the renal tubule. Metabolic acidosis drives the above reaction to the right and increases the plasma concentration of HS, thereby promoting diffusion across the blood-brain barrier into the CNS. In patients with salicylate intoxication, the beneficial effects of sodium bicarbonate are mediated by the production of metabolic alkalosis that decreases the amount of lipid soluble salicylate and driving the above reaction to the left resulting in decreased penetration into central nervous system and in increased urinary clearance [56]. As was discussed above, IV sodium bicarbonate may have serious undesired effects including hypokalemia. The development of hypokalemia is particularly detrimental to the patients with salicylate-mediated toxicity because of increase in hydrogen ion urinary excretion resulting in more acidic urine and greater salicylate reabsorption and thus, should be aggressively corrected [55, 56]. It is important to note that mild alkalemia from a respiratory alkalosis (arterial pH < 7.55) is not a contraindication to sodium bicarbonate therapy in salicylate poisoning. There is no scientifically validated dosing of IV sodium bicarbonate (as in the management of sodium channel blocker toxicities) but it is typically dosed as 1-2 mEq/kg initially administered in bolus doses (see Table 1) and then may be administered as a continuous IV infusion after dilution in dextrose 5% solution [56]. The infusion rate should be titrated to target a urine pH 7.5–8. Blood gas analysis every two hours is indicated for monitoring to prevent severe alkalemia (arterial pH > 7.60) [55, 56]. Patients with anuric renal failure should not receive IV sodium bicarbonate but rather be evaluated for renal replacement therapy [55].

## 5. The Role of Sodium Bicarbonate in the Management of Methanol and Ethylene Glycol Poisoning

Methanol and ethylene glycol are alcohols commonly used in household solutions such as various cleaners, solvents, machine fluids, and antifreeze solutions [57]. There were 52430 exposures to alcohols resulting in 174 fatalities in 2013 [55]. The majority of toxicities arise either as a result of a suicidal attempt or after drinking the toxic alcohol as a substitute for ethanol [57].

**Figure 3:**
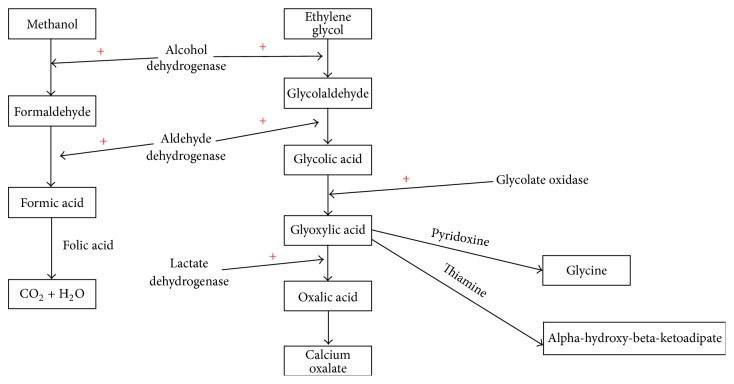
An overview of in vivo methanol and ethylene glycol metabolism.

To understand the basic pathogenesis of methanol and ethylene glycol toxicity, it is important to review briefly the metabolism in vivo. When ingested, both methanol and ethylene glycol undergo an initial biochemical reaction catalyzed by alcohol dehydrogenase (the same enzyme metabolizing ethanol) converting the parent alcohol into formaldehyde and glycolaldehyde, respectively. The metabolism of methanol and ethylene glycol disrupts cellular energy metabolism leading to cellular damage [58, 59]. The final products of methanol and ethylene glycol metabolism are formic acid and oxalic acid, respectively [57]. These end products result in classic features of toxicity such as retinal toxicity caused by methanol and renal injury mediated by oxalic acid. A brief sketch of the in vivo methanol and ethylene glycol metabolism is presented in Figure 3.

Clinical presentations of methanol and ethylene glycol overlap, including central nervous system (CNS) symptoms such as headache, altered mental status, and seizures. Retinal toxicity and blindness are more specific for methanol; acute kidney injury and hypocalcemia are more typical for ethylene glycol intoxication. Laboratory testing and diagnosis are based on the presence of high anion gap metabolic acidosis, the presence of a serum osmolal gap (the difference between measured and calculated osmolality > 10), and measuring the levels of toxic alcohols (used for confirmation; typically, this testing is not time sensitive, and the treatment should not be withheld in any patient suspected of having toxic alcohol ingestion).

As in the management of other discussed toxidromes, the literature on the benefits of IV sodium bicarbonate originates from case reports and consensus guidelines [58, 59]. Beneficial effects of sodium bicarbonate in the management of methanol and ethylene glycol poisoning are believed to be secondary to the formation and enhanced urinary clearance of less toxic metabolites (formate) [59]. Acidemia leads to protonation of methanol and ethylene glycol metabolites to uncharged molecules (e.g., formic acid and oxalic acid), making them more likely to penetrate end-organ tissues (such as the retina) and more likely to be reabsorbed across the renal epithelium from the urine [58]. The suggested regimen for IV sodium bicarbonate is similar to the above-discussed indications. However, according to the consensus guidelines, the therapy with IV sodium bicarbonate should be strongly considered when pH falls below 7.3 with the therapeutic aim of pH normalization. As is the case with any treatment involving IV sodium bicarbonate, administration necessitates frequent monitoring of metabolic parameters (serum electrolytes and renal function), cardiopulmonary, and renal status of the patient. Administration of sodium bicarbonate should be used with caution in a patient with oliguric/anuric renal failure (which is classically seen in severe ethylene glycol toxicity). It is important to note that symptomatic hypocalcemia should be corrected (the routine correction of asymptomatic hypocalcemia is discouraged because of the possible increase in the formation of calcium oxalate crystals in ethylene glycol toxicity).

## 6. The Role of Sodium Bicarbonate in the Management of Miscellaneous Drug Toxicities

IV administration of sodium bicarbonate may result in enhanced urinary excretion of certain chemicals through urinary alkalinization [56]. The urinary clearance of methotrexate, phenobarbital, chlorpropamide, and fluoride is increased after reaching urinary pH levels of 7.5–8.0 via formation of lipid insoluble metabolite of the parent drug.

Metformin toxicity is another instance where sodium bicarbonate may be used [60]. In rare circumstances (such as acute illness and worsening renal function), metformin administration may result in the development of lactic acidosis that is believed to be secondary to mitochondrial dysfunction, with a shift towards anaerobic glycolysis [60–62]. Though the literature on the role of systemic bicarbonate administration in cases of metformin poisoning is very scant, the use of IV sodium bicarbonate should be probably limited to nonanuric patients with metformin-associated advanced metabolic acidosis and pH < 7.1, given the questionable efficacy and potential for adverse effects.

In conclusion, the data on the role of sodium bicarbonate in the management of the above-listed medications is even more limited and cannot be recommended as a first line.

## 7. Conclusion

Based on the available literature and empiric experience, the IV administration of sodium bicarbonate appears to be beneficial in the management of certain pharmacological toxicities such as sodium channel blockers poisonings, salicylate intoxication, and ingestion of methanol and ethylene glycol. However, most of the data originates from case reports, case series, and expert consensus recommendations. The data on the management of metformin-associated lactic acidosis, chlorpropamide, methotrexate, and phenobarbital is even more limited. However, it seems very unlikely that randomized controlled trials assessing the impact of sodium bicarbonate will be performed due to ethical concerns.

The potential mechanisms of sodium bicarbonate administration include high sodium load, development of metabolic alkalosis with resultant decreased tissue penetration of the toxic substance, and its increased urinary excretion, while, on IV sodium bicarbonate, the patients must be monitored for the development of associated side effects including electrolyte abnormalities (hypokalemia, hypocalcemia, and hypernatremia), progression of metabolic alkalosis volume overload, worsening respiratory status (volume overload and increased CO_2_ production), and worsening metabolic acidosis (paradoxical increase in lactic acid production secondary to the activation of glycolytic enzymes). Frequency of laboratory testing should be individualized. Blood gas analysis (arterial or venous) and chemistry tests should be monitored at least every 4 hours or more frequently if clinically indicated in patients treated with sodium bicarbonate. It is important to keep calcium and potassium within normal range to decrease the risk of cardiac arrhythmias.

Patients with oliguric/anuric renal failure and advanced decompensated heart failure should not receive IV sodium bicarbonate. IV administration of sodium bicarbonate represents only one aspect of the complex management of medication and chemical toxicities, and, for a thorough discussion of the management of these toxicities, the reader is referred to focused reviews.

## Figures and Tables

**Table 1:** Constituents and characteristics of commonly used crystalloids and plasma.

Solution	Ph	Na^+^ (mmol/l)	Cl^−^ (mmol/l)	K^+^ (mmol/l)	Osmolality (mOsmol/kg)
Plasma	7.35–7.45	136–145	95–105	3.5–5.0	275–295
Lactated Ringer	6.6	130	109	4	273
3% sodium chloride	5.0	513	513	0	1027
Normal saline (0.9%)	5.7	154	154	0	308
Half-normal saline (0.45%)	5.6	77	77	0	154
Dextrose 5% in water (D5W)	4.5	0	0	0	253
Bicarbonate (8.4%)^*∗*^	8	1000	0	0	2000

^*∗*^1 ampule contains 50 milliliters with 50 mEq of sodium bicarbonate.

**Table 2:** List of some drugs with sodium channel blocking properties.

Class	Representative medications
Class Ia antiarrhythmics	Quinidine, Procainamide
Class Ib antiarrhythmics	Lidocaine, Phenytoin
Class Ic antiarrhythmics	Propafenone, Flecainide
Class III antiarrhythmics	Amiodarone, Sotalol
Tricyclic antidepressants	Amitriptyline, Doxepin
Antiepileptic medications	Carbamazepine, Lamotrigine, Zonisamide, Lacosamide
Selective serotonin reuptake inhibitors	Citalopram, Venlafaxine, Fluoxetine
Antihistamines	Diphenhydramine
Miscellaneous	Cocaine, local anesthetics, Thioridazine, Propranolol, Amantadine, Chloroquine, Hydroxychloroquine, Cyclobenzaprine

## Abbreviations

CNS: Central nervous system
ECG: Electrocardiogram
IV: Intravenous
TCA: Tricyclic antidepressant
USA: United States of America.
